# Therapeutic potential of mesenchymal stem cells and their exosomes in severe novel coronavirus disease 2019 (COVID-19) cases

**DOI:** 10.1186/s41232-020-00121-y

**Published:** 2020-06-22

**Authors:** Atsunori Tsuchiya, Suguru Takeuchi, Takahiro Iwasawa, Masaru Kumagai, Takeki Sato, Satoko Motegi, Yui Ishii, Youhei Koseki, Kei Tomiyoshi, Kazuki Natsui, Nobutaka Takeda, Yuki Yoshida, Fusako Yamazaki, Yuichi Kojima, Yusuke Watanabe, Naruhiro Kimura, Kentaro Tominaga, Hiroteru Kamimura, Masaaki Takamura, Shuji Terai

**Affiliations:** Division of Gastroenterology and Hepatology, Graduate School of Medical and Dental Science, Niigata University, 1-757 Asahimachi-dori, Chuo-ku, Niigata, 951-8510 Japan

**Keywords:** COVID-19, SARS-CoV-2, Mesenchymal stem cells, Exosome, Cytokine storm, Acute respiratory distress syndrome

## Abstract

The novel coronavirus severe acute respiratory syndrome coronavirus 2 (SARS-CoV-2) is the causative agent of coronavirus disease 2019 (COVID-19) and the ensuing worldwide pandemic. The spread of the virus has had global effects such as activity restriction, economic stagnation, and collapse of healthcare infrastructure. Severe SARS-CoV-2 infection induces a cytokine storm, leading to acute respiratory distress syndrome (ARDS) and multiple organ failure, which are very serious health conditions and must be mitigated or resolved as soon as possible. Mesenchymal stem cells (MSCs) and their exosomes can affect immune cells by inducing anti-inflammatory macrophages, regulatory T and B cells, and regulatory dendritic cells, and can inactivate T cells. Hence, they are potential candidate agents for treatment of severe cases of COVID-19. In this review, we report the background of severe cases of COVID-19, basic aspects and mechanisms of action of MSCs and their exosomes, and discuss basic and clinical studies based on MSCs and exosomes for influenza-induced ARDS. Finally, we report the potential of MSC and exosome therapy in severe cases of COVID-19 in recently initiated or planned clinical trials of MSCs (33 trials) and exosomes (1 trial) registered in 13 countries on ClinicalTrials.gov.

## Background

In Wuhan, China, an outbreak of pneumonia of an unknown cause was reported in December 2019. In January 2020, a novel coronavirus, termed severe acute respiratory syndrome coronavirus 2 (SARS-Co V-2), was isolated from the patient samples [1, 2]. In February 2020, the infection was designated as coronavirus disease 2019 (COVID-19) by the World Health Organization (WHO). Since then, COVID-19 has rapidly spread to more than 200 countries, which have experienced activity restriction, economic stagnation, and collapse of the healthcare system to varying degrees [3]. The median incubation period in some patients is quite long (5.0 days; confidence interval 4.4 to 5.6 days), with the incubation period ranging from 2 to 14 days [4, 5]. While some patients show very mild symptoms and may even be asymptomatic, the elderly, and those with chronic diseases such as lung disease and diabetes mellitus often progress to a severe case of acute respiratory distress syndrome (ARDS) and ultimately suffer multiple organ failure (MOF) with a high-mortality rate [1, 2, 6–8]. In addition, very few existing anti-viral drugs have shown therapeutic effect for this virus. These characteristics of COVID-19 make it a challenging disease to control. Thus, a multidirectional approach from prevention to treatment is warranted. Currently available drugs that can target the viral replication cycle have attracted attention. For example, the following three drugs have been considered for COVID-19: camostat mesylate, which inhibits protein-mediated fusion of the virus with cell membranes; favipiravir, which is an anti-viral drug targeting influenza viruses; and remdesivir, which is an anti-viral drug originally developed against Ebola virus [9]. Thus, drugs that not only target the replication cycle, but also prevent and treat the cytokine storm observed in severe cases of COVID-19, need to be discovered. For the severe COVID-19 cases with cytokine storms, mesenchymal stem cells (MSCs) and their exosomes are a potential treatment option [10–12]. In this review, we discuss the therapeutic potential of MSCs and their exosomes for severe COVID-19 cases.

## Presentation of severe cases of COVID-19

The presentation of severe cases of COVID-19 is currently under investigation. It was reported that the median time from first symptom to dyspnea was 5.0 days, to hospital admission was 7.0 days, and to ARDS was 8.0 days [7]. Studies from China and the USA reported that 14.1% and 12.1% of patients with COVID-19 were in severe condition at the time of admission in Wuhan and New York, respectively [13, 14]. Lung images, acquired by X-ray or computed tomography, together with hematological and biochemical blood parameters such as elevation of neutrophil count, D-dimer, alanine aminotransferase, total bilirubin, lactate dehydrogenase, ferritin and procalcitonin, prolonged prothrombin time, and decreases in lymphocyte counts and albumin have been reported in severe cases [2, 15, 16]. During the process of aggravation, virus-induced cytopathic effects and viral evasion of host immune responses are believed to dictate disease severity. In a previous human coronavirus study, it was reported that robust viral replication with delayed interferon (IFN) response causes aggressive infiltration of monocytes/macrophages and neutrophils, and very high production of cytokines and chemokines [10]. A study of subjects who died of Middle East Respiratory Syndrome (MERS) and SARS suggests that an aberrant host immune response results in an inflammatory cytokine storm (also called cytokine release syndrome (CRS), macrophage activation syndrome (MAS), or secondary hemophagocytic lymphohistiocytosis (sHLH), followed by ARDS and MOF [10, 17]). A subject who died of severe COVID-19 with cytokine storm showed tissue necrosis and interstitial macrophage and monocyte infiltration in the lungs, heart, and gastrointestinal mucosa. In severe cases, as discussed above, severe lymphocytopenia, with hyperactivated T cells and decreased numbers of regulatory T cells, is commonly observed [18–20]. Elevated cytokine and chemokine levels are being reported in COVID-19 cases. Specifically, the levels of IFN-γ, interleukin (IL)-1β, 2, 4, 6, 8, 10, and 17, induced protein 10 (IP10), monocyte chemoattractant protein-1 (MCP-1), granulocyte-colony stimulating factor (G-CSF), and tumor necrosis factor α (TNFα) are significantly elevated in patients with severe COVID-19, compared to non-severe cases [1, 2, 6, 7, 21]. Chen et al. further reported that increased levels of the cytokines IL-6, IL-10, and TNF-α, lymphocytopenia of CD4^+^ and CD8^+^ T cells, and decreased IFN-γ expression in CD4^+^ T cells are associated with severe COVID-19 [16]. These results suggest that a dysregulated immune response contributes to the aggravation of the disease. Of these cytokines and chemokines, IL-6, which is produced by pathogenic T cells and monocytes, has attracted attention as a therapeutic target for severe cases of COVID-19. Xu et al. reported that the therapeutic effect of tocilizumab, a recombinant humanized anti-human IL-6 receptor monoclonal antibody, could improve patient outcomes without apparent adverse events including secondary infection [22]. All of these results suggest that MSCs, which possess an immunomodulatory ability, have the potential to be effective in decreasing or treating severe cases, in addition to anti-viral drugs.

## Administration of MSCs and their exosomes as candidate therapy for the prevention of aggravation and treatment of severe COVID-19 cases

MSCs were originally defined to fulfill the following three minimum criteria postulated by the International Society for Cellular Therapy. First, MSCs must be plastic-adherent when maintained under standard culture conditions. Second, they must express CD105, CD73, and CD90, and lack the expression of CD45, CD34, CD14 or CD11b, CD79a, or CD19, and HLA (human leucocyte antigen)-DR surface molecules. Third, MSC must differentiate into osteoblasts, adipocytes, and chondroblasts in vitro [23]. These cells are relatively easy to expand, maintain, and cryopreserve, while maintaining their viability. MSCs can be obtained not only from bone marrow, but also from medical waste such as umbilical cord tissue, adipose tissue, amniotic tissue, and dental pulp. A recent study reported that MSCs with different tissue origins have somewhat different characteristics [24]. However, overall, MSCs express low or modest levels of major histocompatibility complex (MHC) class I molecules and lack the expression of MHC class II and co-stimulatory molecules, such as CD40, CD80, and CD86, leading to their low immunogenicity. This suggests that MSCs can avoid immune responses in recipients. Thus, autologous or allogeneic MSCs have been used in clinical studies [25–28]. The main functions of MSCs are determined by trophic factors including chemokines, cytokines, growth factors, and exosomes. Watanabe et al. reported in a mouse study that most MSCs injected into the tail vein migrated to the lung, with a small proportion migrating to the liver. MSCs exhibit anti-inflammatory, anti-fibrotic, and anti-oxidant effects, and can promote angiogenesis [29]. Therefore, they have been used in many fields to treat a variety of diseases such as neural, heart, liver, intestinal, and lung diseases, among others. According to ClinicalTrials.gov, more than 900 clinical trials involving MSCs have been registered [27]. In addition, MSCs have higher immunomodulatory/immunosuppressive effects when the host inflammatory status is hyperactive [30, 31]. For example, the allogeneic mesenchymal stromal cell-based agent TEMCELL® was approved for treating grade III or IV acute graft-versus-host disease (GVHD) in Japan [32]. A variety of effects of MSC therapy on immune cells, including their ability to induce anti-inflammatory macrophages, regulate T and B cells and regulatory dendritic cells, and inactivate T cells have been reported. Thus, MSCs, which can elicit multidirectional therapeutic effects, can be theoretically appropriate for treating severe COVID-19 cases [29–31, 33].

**Fig. 1:**
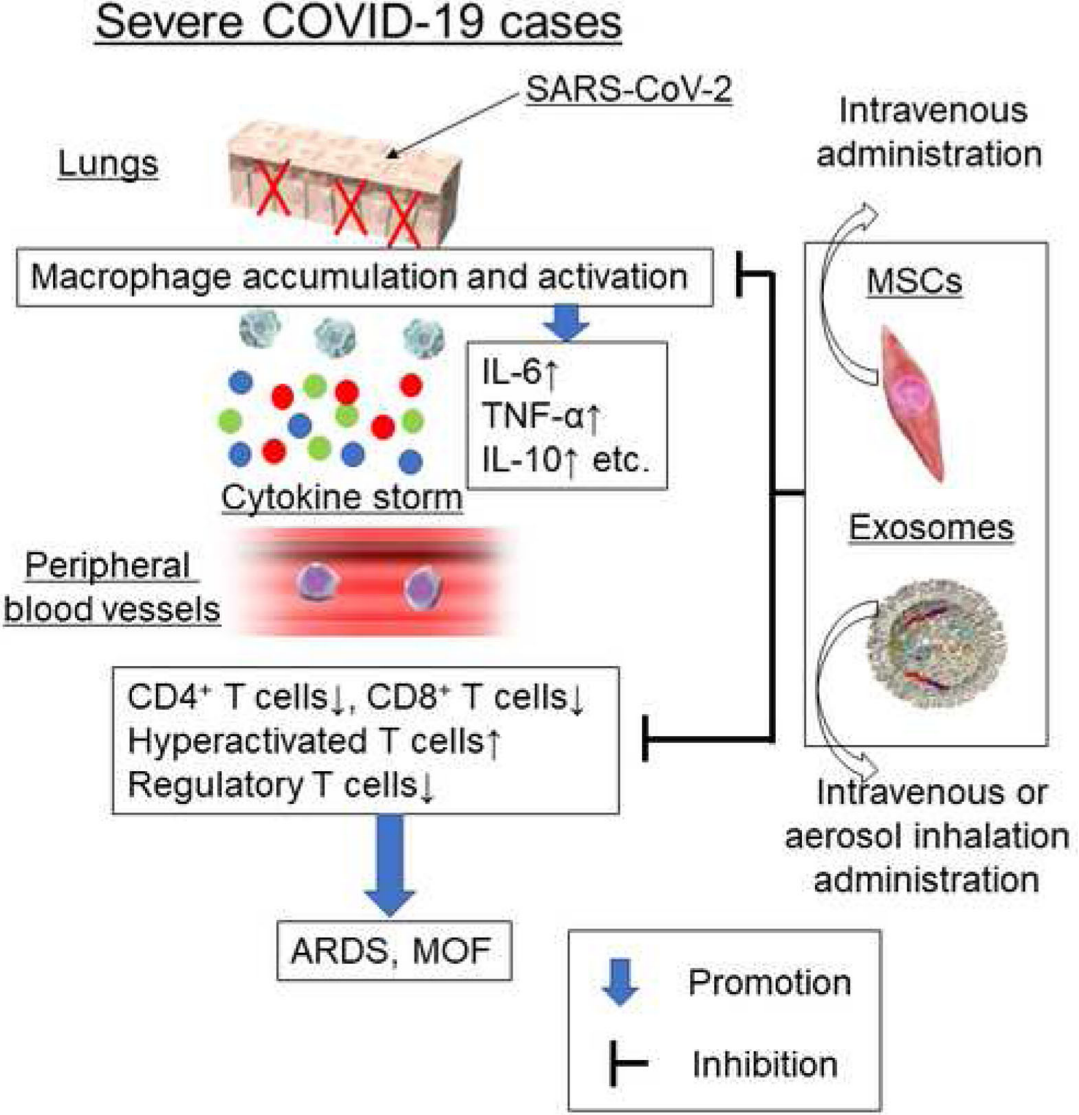
Putative mechanisms of mesenchymal stem cell (MSC) and exosome therapy in severe COVID-19 cases. MSCs and their exosomes have a potent ability to ameliorate the cytokine storm observed in severe cases of COVID-19, and prevent progression toward acute respiratory distress syndrome (ARDS) and multiple organ failure (MOF). SARS-CoV-2, severe acute respiratory syndrome coronavirus 2; IL, interleukin

Exosomes (or small extracellular vesicles) produced by MSCs have recently attracted attention because they are thought to be important for MSC activity. Exosomes are approximately 100-nm wide and are formed by the endosomal system of the cell. They are enclosed by a lipid bilayer and are thus, very stable and harbor molecules including proteins, RNAs, lipids, and metabolites. In addition, MSC-derived exosomes possess hypoimmunogenic properties similar to MSCs. Theoretically, exosomes may also serve as an effective therapeutic option for various diseases. Exosomes are difficult to collect and enrich; thus, techniques to harvest them are currently being optimized. In some studies, exosomes have been shown not to be trapped in the lungs like MSCs but instead, migrate to the target organ. Thus, they may be advantageous for therapeutic applications via aerosol inhalation [34–39] (Fig. 1).

## MSCs for severe influenza and COVID-19

A basic study using an influenza model and a clinical study on influenza A and COVID-19 showed promising results. With regard to the basic study on an influenza model, Loy et al. reported the therapeutic efficacy of human umbilical cord tissue-derived MSCs (UC-MSCs) using a model of acute lung injury (ALI) induced by the influenza A (H5N1) virus. They confirmed that UC-MSCs were effective in restoring impaired alveolar fluid clearance and protein permeability of influenza A-infected alveolar epithelial cells. They also confirmed that conditioned UC-MSC medium and UC-MSC exosomes have some therapeutic effects and only UC-MSCs slightly improved the survival of influenza A-infected mice [40]. In a clinical study on influenza using MSCs, Chen et al. enrolled 44 patients with H7N9-induced ARDS as a control group and 17 patients with H7N9-induced ARDS as the MSC injection group. They used allogeneic, menstrual-blood-derived MSCs from a healthy female donor (age 20–45). They reported that three patients were treated with three infusions of MSCs at the early stage of H7N9 infection, while the other six patients were treated with three infusions of MSCs at the late stage of H7N9 infection, and eight patients received four infusions of MSCs at the late stage of H7N9 infection; the injection dose of MSCs was 1 × 10^6^/kg each time. No MSC-infusion-related acute toxicities or seriously adverse events were observed in any of these patients. A multiple intravenous infusion of MSCs was tolerated in these patients with moderate to severe H7N9-induced ARDS. They also reported that MSC transplantation significantly lowered the mortality of the subjects in the experimental group compared with that in the control group (17.6% died in the experimental group, while 54.5% died in the control group) [41]. These results showed that MSCs have therapeutic effects on ARDS and may have a similar effect on patients with COVID-19. With regard to the clinical study on severe COVID-19 cases, Leng et al. reported that seven cases (two common, four severe, and one critically severe case) were treated with MSCs. They concluded that MSCs significantly improved the functional outcome of all seven patients without any adverse effects. The pulmonary function of these seven patients significantly improved 2 days after MSC injection. Among them, two subjects with common COVID-19 and one subject with severe COVID-19 recovered and were discharged 10 days after treatment [42]. Although the number of COVID-19 patients who underwent MSC treatment is very limited and there is a lack of studies elucidating the underlying mechanisms, this study shows the potential application of MSC therapy for severe COVID-19 cases.

## Recently started or planned clinical trials of MSCs and exosomes

Clinical trials using MSCs and exosomes have already begun or are planned. To describe recent trends in clinical trials using MSCs, we evaluated clinical studies reported on ClinicalTrials.gov. We searched the words “COVID,” “mesenchymal,” or “exosome” and identified that 33 MSC trials and one exosome trial have been registered. As shown in Additional file 1: Table S1, of the 33 trials, there were 10 (30.3 %) from China, 8 (24.2%) from the USA, 4 (12.1%) from Spain, and 1 (3.0%) each from Brazil, UK, Jordan, France, Denmark, Iran, Columbia, Germany, Turkey, and Canada. Thirteen trials (39.4%) used UC-MSCs, 6 trials (18.2%) used bone marrow-derived MSCs, five trials (15.1%) used adipose tissue-derived MSCs and two trials (6.1%) used dental pulp-derived MSCs. Twenty-six trials (78.8%) used allogeneic MSCs and one trials (3.0%) used autologous MSCs. Intravenous administration (1–5 injections) was performed in all trials and the dose was approximately 1 × 10^6^ cells/kg. Almost all trials are phase I, II, or I/II studies (One trial is phase II/III). A pilot study using MSC-derived exosomes for treating severe COVID-19 has been registered in China. They plan to administer 2.0 × 10^8^ nanovesicles five times by aerosol inhalation. While basic studies using MSCs and exosomes have not been sufficiently performed for COVID-19, clinical studies using MSCs and exosomes are in the planning stage or recently initiated (Additional file 1: Table S1).

## Conclusions

To combat the COVID-19 pandemic, we must establish a multidirectional approach to reduce its prevalence. For the prevention of aggravation, and for the treatment of severe cases of COVID-19, MSC and exosome therapy may be a potential option. However, in parallel, we need to perform further experiments to understand the underlying mechanism of action of SARS-CoV-2 in more detail and accordingly optimize MSC therapy, including parameters such as culture conditions and pre-conditioning. To prepare for the prolonged COVID-19 pandemic or other such pandemics, we must explore novel approaches, such as development of exosome therapy, by using biologically active molecules.

## Supplementary information

**Additional file 1: Table S1.** Clinical trials using mesenchymal stem cells (MSCs) and exosomes and registered on ClinicalTrials.gov.

## Data Availability

Not applicable.

## Abbreviations

MSC: Mesenchymal stem cell
SARS-CoV-2: Severe acute respiratory syndrome coronavirus 2
COVID-19: Coronavirus disease 2019
ARDS: Acute respiratory distress syndrome
MOF: Multiple organ failure
IFN: Interferon
MERS: Middle East Respiratory Syndrome
CRS: Cytokine release syndrome
MAS: Macrophage activation syndrome
sHLH: Secondary hemophagocytic lymphohistiocytosis
IP10: Induced protein 10
MCP-1: Monocyte chemoattractant protein-1
G-CSF: Granulocyte-colony stimulating factor
TNFα: Tumor necrosis factor α
HLA: Human leucocyte antigen
MHC: Major histocompatibility complex
GVHD: Graft-versus-host disease
ALI: Acute lung injury
